# Using Tractography to Distinguish SWEDD from Parkinson's Disease Patients Based on Connectivity

**DOI:** 10.1155/2016/8704910

**Published:** 2016-02-29

**Authors:** Mansu Kim, Hyunjin Park

**Affiliations:** ^1^Department of Electronic, Electrical and Computer Engineering, Sungkyunkwan University, Suwon 16419, Republic of Korea; ^2^School of Electronic and Electrical Engineering, Sungkyunkwan University, Suwon 16419, Republic of Korea; ^3^Center for Neuroscience Imaging Research (CNIR), Institute for Basic Science, Suwon 16419, Republic of Korea

## Abstract

*Background*. It is critical to distinguish between Parkinson's disease (PD) and scans without evidence of dopaminergic deficit (SWEDD), because the two groups are different and require different therapeutic approaches.* Objective*. The aim of this study was to distinguish SWEDD patients from PD patients using connectivity information derived from diffusion tensor imaging tractography.* Methods*. Diffusion magnetic resonance images of SWEDD (*n* = 37) and PD (*n* = 40) were obtained from a research database. Tractography, the process of obtaining neural fiber information, was performed using custom software. Group-wise differences between PD and SWEDD patients were quantified using the number of connected fibers between two regions, and correlation analyses were performed based on clinical scores. A support vector machine classifier (SVM) was applied to distinguish PD and SWEDD based on group-wise differences.* Results*. Four connections showed significant group-wise differences and correlated with the Unified Parkinson's Disease Rating Scale sponsored by the Movement Disorder Society. The SVM classifier attained 77.92% accuracy in distinguishing between SWEDD and PD using these identified connections.* Conclusions*. The connections and regions identified represent candidates for future research investigations.

## 1. Introduction

Parkinson's disease (PD), the second most common neurodegenerative disease in the elderly, causes the loss of dopaminergic neurons in the brainstem, especially those in the substantia nigra [1]. Characteristic PD symptoms include resting tremor, rigidity, impaired voluntary movement, and cognitive impairment [2]. PD is typically diagnosed by criteria such as those of the United Kingdom's Parkinson's Disease Society Brain Bank [3]. However, about 10% of clinically diagnosed PD patients do not lose dopaminergic neurons in the brainstem and are classified as scans without evidence of dopaminergic deficit (SWEDD) [4]. SWEDD is a nuclear medicine term referring to patients with PD that show no evidence of dopamine transporter deficit. The etiology of SWEDD has been under debate; some consider it an early phase of PD, while others consider it a different disease [5]. Distinguishing between PD and SWEDD is important, as the two groups are potentially different and thus may require different therapeutic strategies.

Diffusion tensor imaging (DTI), an MRI imaging technique, quantifies* in vivo* neuronal fiber information using anisotropic water diffusion in the white matter. This approach has been used to differentiate between normal controls (NCs) and PD patients [6–8]. DTI has a few limitations and is unable to distinguish between efferent and afferent connections; however, it is the only practical option for assessing* in vivo* fiber information [9]. Raw data from DTI need to be processed with an algorithm in a process known as tractography so that relevant fiber information can be extracted [10]. Extracted fiber information is further analyzed with connectivity analysis, which is suitable for analyzing complex objects such as the brain. Connectivity derived from DTI is known as structural connectivity, because DTI is related to actual neuronal fiber connections between regions. Many studies have also derived brain connectivity information from resting state functional MRI (fMRI), which measures local brain activity based on the blood oxygen level-dependent (BOLD) effect [11].

Connectivity analysis requires that regions of interest (ROIs) are specified in order to explore correlations or connections among the regions. These ROIs can be specified by coregistering information from a predefined parcellation of the brain, which can be derived from structural or functional information [12]. Brain connectivity has been explored for analysis of motor-related regions previously known to be affected by PD [13]. Structural connectivity of cortico-cortical, cortical-subcortical, and subcortical connections in cortico-basal ganglia-thalamocortical (CBTC) connections was investigated in this study. Connectivity measures can be used to quantify group-wise differences and as biomarkers of important clinical variables in PD research. Many studies have successfully established group-wise differences between PD patients and NCs based on neuroimaging [2, 6–8, 13–15]. However, less attention has been paid to differences between PD and SWEDD patients. Thus, the focus of this study was to quantify differences between PD and SWEDD patients based on connectivity information from DTI tractography.

We obtained diffusion MRI data from the Parkinson's disease Progressive Marker Institute (PPMI) research database [16]. Connectivity analysis was applied to PD and SWEDD patients. Group-wise differences between PD and SWEDD patients were quantified using the number of neuronal fibers connecting regions as features. We tried to identify connections between regions with significant group-wise differences in terms of the number of neuronal fibers and then find significant correlations between the number of fibers that connected specific regions and the Movement Disorder Society-sponsored Unified Parkinson's Disease Rating Scale (MDS-UPDRS).

## 2. Material and Methods

### 2.1. Subjects

Our study was a retrospective analysis of anonymized imaging data, and local IRB approval was obtained. The PPMI is an observational clinical study to evaluate PD and other closely related cohorts using advanced imaging, biological sampling, and clinical and behavioral assessments to identify potential biomarkers of progression of PD [16]. The PPMI includes a database where imaging and clinical/behavioral assessments data are shared with the scientific community. The study included 423 de novo PD, 64 SWEDD, and 196 normal control cases as of July 2015. Our study explored subjects with MRI imaging data aside from the usual SPECT. Our SWEDD group was limited to 37 SWEDD patients who underwent DTI and structural MRI imaging. We included a total of 77 participants and classified them into SWEDD (*n* = 37) and PD (*n* = 40) groups that were matched according to age and sex ratio. Subgroups were classified based on PPMI consortium guidelines [16]. Detailed criteria for the SWEDD group are as follows. First, patients had to demonstrate at least two of the following symptoms: resting tremor, bradykinesia, rigidity, or either asymmetric resting tremor or bradykinesia. Second, patients had to have been diagnosed with PD for 2 years or less at the time of screening, with no evidence of dopamine transporter deficit on dopamine transporter SPECT imaging. Third, patients had to have been free of PD medication for at least 6 months. Fourth, patients were 30 years or older at the time of PD diagnosis. Participant information, including scores of MDS-UPDRS, Montreal Cognitive Assessment (MoCA), and Geriatric Depression Scale (GDS), is given in Table 1. The disease duration of all (*n* = 422) available PD patients in the PPMI database was 6.57 ± 6.51 months (mean ± SD), which was not significantly different from the PD group used in our study (*P* = 0.343). The imaging data of PD and SWEDD were from baseline scans of patients not using any PD-related medication.

### 2.2. Imaging Data

We obtained diffusion and T1- and T2-weighted MRI data from the PPMI database [16]. We obtained T1- and T2-weighted MR imaging in addition to the DTI data because the image preprocessing steps were required in T1- and T2-weighted MR imaging. T1- and T2-weighted MRI data were obtained using the following parameters: TR = 2300 ms, TE = 2.98 ms, TI = 900 ms, image matrix = 240 × 256 × 176, and voxel resolution = 1 × 1 × 1 mm^3^. Parameters for the T2-weighted images were as follows: TR = 3000 ms, TE = 101 ms, image matrix = 228 × 256 × 48, and voxel resolution = 0.9375 × 0.9375 × 3 mm^3^. Diffusion images were obtained with a Siemens 3T scanner using the following parameters: 3T scanner, *b* = 1000 s/mm^2^, 64 diffusion gradient directions with one *b*0 image, image matrix = 116 × 116 × 72, and voxel resolution = 1.98 × 1.98 × 2 mm^3^.

### 2.3. Image Preprocessing

**Figure 1:**
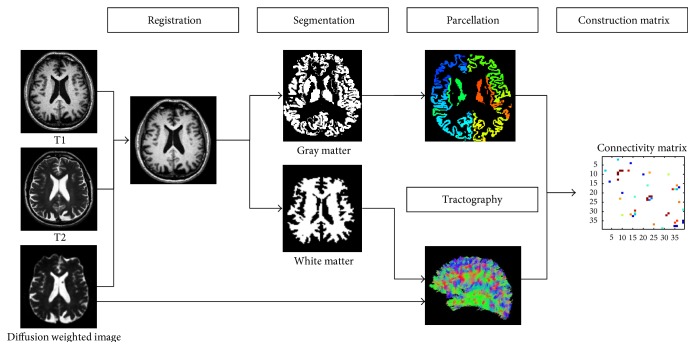
Overall image preprocessing procedures.

Preprocessing steps were performed to extract fiber information from DTI data. Readers interested in details of the preprocessing steps are referred to a review article [17]. Briefly, image preprocessing was performed using the Connectome Mapping Toolkit (CMTK), a Python-based open-source software (http://www.cmtk.org/). T1- and T2-weighted images were aligned to the non-diffusion-weighted image (*b*0) by a nonlinear registration using FSL for each subject [18]. The registered T1-weighted image was segmented into white matter, grey matter, and cerebrospinal fluid using FreeSurfer [19]. The segmented white matter was then used to guide the tractography algorithm. An overview of image preprocessing procedures is provided in Figure 1.

### 2.4. ROI Specifications

Exploring connectivity requires that the ROIs are specified so that correlations among them can be investigated. We coregistered and transferred information from a predefined atlas onto the individual subject image spaces to specify the ROIs using the Desikan-Killiany anatomical atlas [12]. Our analysis focused on the 39 motor-related regions known to be affected by PD. A full list of explored regions is provided in Table 2 [13]. The regions include the motor cortex, premotor cortex, dorsal lateral prefrontal cortex (DLPFC), cingulate motor area, supplementary motor area (SMA), and a few regions in the parietal lobe, subcortex, and brainstem. The cortico-cortical connections mediate hand, face, foot, and trunk motor activity [20]. The CBTC connection has been shown to be important in PD patients [21]. Fiber connections from the brainstem were included to explore the initiation and control of movement [22]. Connections from the brainstem were considered because this structure is densely connected with the pallidum and substantia nigra, which are important regions affected by PD. Connections among these regions were explored to quantify structural connectivity of cortico-cortical, cortical-subcortical, and subcortical connections.

### 2.5. Tractography and Network Construction

Eddy current-induced distortions and head motion were corrected with FSL [18]. Fiber information was computed using the FACT algorithm implemented within the Diffusion Toolkit and CMTK software [17]. The FACT algorithm propagated a line from the center of a seed voxel along the direction of the dominant vector, which was determined by the largest eigenvector of the tensor. The starting point of the next voxel was the intercept of the previous voxel. Tracking terminated when the algorithm reached a region with an abrupt change in fiber direction (i.e., angle threshold greater than 60°). Tracking was limited to white matter and its neighbors, as neuronal fibers mainly exist in the white matter. Every voxel was considered a seed voxel, and we retained only fibers whose end points were within the predefined ROIs.

Connectivity was assessed using nodes and edges of a graph. Nodes were assigned ROIs that were transferred from the predefined parcellation. Each edge value was assumed to be the number of fibers whose end points resided within two ROIs, which was entered into the matrix as an element. Elements with less than 5 fibers were excluded, as identification of a very small number of fibers was considered to be an unstable result of the tractography algorithm [23]. The matrix was referred to as the connectivity matrix. We adopted a simple network model that considered undirected and weighted edges.

### 2.6. Statistical Tests

Group-wise differences between SWEDD and PD groups were explored in 39 regions. For each group, the connectivity matrices of the two groups (i.e., PD and SWEDD) were stacked into two three-dimensional matrices. Each element in the stacked connectivity matrix contained many observations (i.e., 37 or 40 observations), which were tested using nonparametric permutation tests to distinguish between PD and SWEDD. Permutation tests were performed by randomly assigning PD and SWEDD subjects 10,000 times. One permutation involved randomly assigning the first 37 cases to the SWEDD group and the remaining 40 cases to the PD group. Differences in the number of fibers were deemed significant if they did not belong to the 95th percentile of the null distribution derived from the permutation tests (*P* < 0.05, corrected).

### 2.7. Correlation with Clinical Scores

We performed correlation analysis to detect possible links between structural connectivity and clinical scores. We pooled connectivity matrices between the groups (i.e., PD and SWEDD) into one long vector and then computed Spearman correlations with MDS-UPDRS scores for all identified connections. The Holm-Bonferroni correction was adopted to compute corrected *P* values in order to reveal significantly correlated connections.

### 2.8. Classification Using Identified Connections

The four significant connections were fed into a support vector machine (SVM) classifier framework with linear kernels to separate between SWEDD and PD. The technical details of the SVM are found in a review by Vapnik [24]. We applied the leave-one-out-cross validation method for discrimination of training and test data because of the limited number of available subjects. For example, given 37 SWEDD and 40 PD cases, we assigned one case as the test case and used the remaining 76 cases as the training data for the SVM classifier. The process was repeated 77 times, choosing a different test case each time. The SVM classifier seeks a decision boundary that can effectively separate samples near the decision boundary. Classifier accuracy, sensitivity, and specificity were computed by comparing the classifier outcomes with known ground truth using MATLAB [25].

## 3. Results

### 3.1. Structural Connectivity Differences

**Figure 2:**
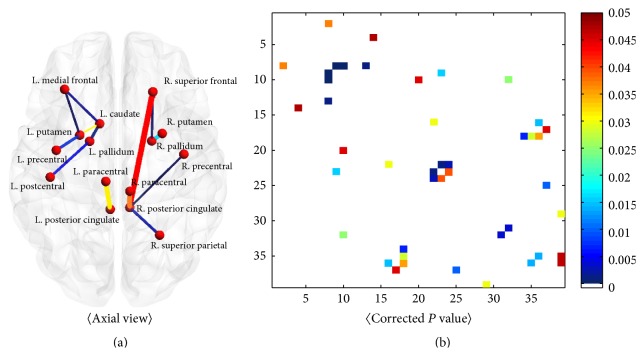
Connections and group-wise differences. Significantly different connections were rendered (a), and corrected *P* values were plotted (b).

The stacked connectivity matrices of SWEDD and PD groups were investigated using nonparametric permutation tests in order to identify elements (i.e., connections between regions) that distinguished between PD and SWEDD patients. Twenty-three connections were significantly different between PD and SWEDD, as shown in Figure 2 and Table 3. Of these connections, 11 increased in PD compared to SWEDD and 12 decreased in PD compared to SWEDD. Overall, eight connections were significant, with corrected *P* values less than 0.01. Of these connections, three had *P* values less than 0.001, including the connections between the paracentral gyrus and posterior cingulate gyrus in the left hemisphere, between the posterior cingulate gyrus and superior frontal lobe in the right hemisphere, and between the posterior cingulate gyrus and paracentral gyrus in the right hemisphere. Further details regarding identified connections are reported in Table 3.

### 3.2. Correlations between Identified Connections and Clinical Scores

**Figure 3:**
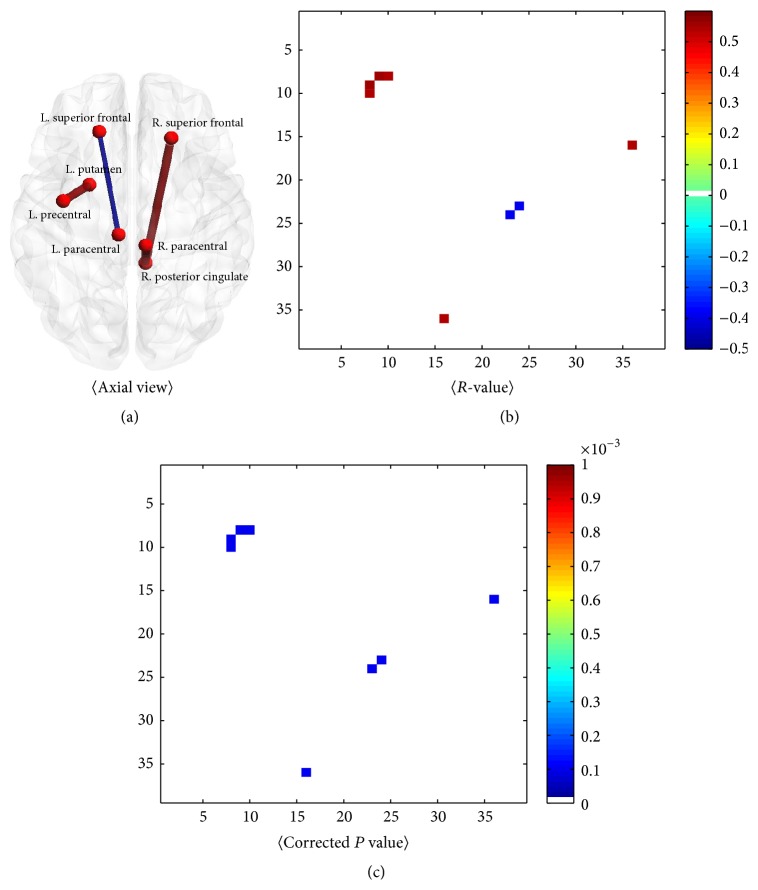
Connections and correlations with MDS-UPDR-III. The significantly correlated connections were rendered (a). *R*-values (b) and corrected *P* values (c) of significantly correlated connections with MDS-UPDRS-III (corrected *P* < 0.001) are plotted.

Correlation analysis was performed to identify possible links between structural connectivity and MDS-UPDRS-III score. The approach was similar to that in the previous section, which sought connections that distinguished two groups, except that, in this case, we sought connections that were significantly correlated with MDS-UPDRS score. Spearman's correlation was adopted because clinical scores are categorical measurements.  Figure 3 shows significantly correlated connections with MDS-UPDRS score in the 39 ROIs. Only a few of the identified connections with group-wise differences were observed to be significantly correlated (corrected *P* < 0.001) with MDS-UPDRS score, as shown in bold text in Table 4. Four connections had significant group-wise differences in structural connectivity and correlation with clinical scores. Clinical scores were correlated with the connection between the precentral gyrus and putamen in the left hemisphere (*r* = 0.54, corrected *P* < 0.001), between the posterior cingulate gyrus and superior frontal lobe in the right hemisphere (*r* = 0.54, corrected *P* < 0.001), between the posterior cingulate gyrus and paracentral gyrus in the right hemisphere (*r* = 0.56, corrected *P* < 0.001), and between the paracentral gyrus and superior frontal lobe in the left hemisphere (*r* = −0.32, corrected *P* < 0.001).

### 3.3. Classifier Performance

The SVM classifier using linear kernels was applied to separate SWEDD and PD cases. Accuracy, sensitivity, and specificity of the classifier are reported in Table 5 for distinguishing between PD and SWEDD cases. Overall, the classification results were good (sensitivity, specificity, and accuracy were 78.38%, 77.50%, and 77.92%, resp.).

## 4. Discussion

We showed that PD and SWEDD patients might be distinguished using connectivity values derived from fiber connections. The four connections that distinguished between PD and SWEDD also correlated well with clinical scores, indicating their relevance. Furthermore, our study successfully classified between PD and SWEDD using these identified connections. This was a proof of concept study, however, and our findings should be interpreted cautiously in terms of clinical application. Further research with more samples is needed to validate our findings.

Among cortico-cortical connections, bilateral paracentral-posterior cingulate, precentral-posterior cingulate, and posterior cingulate-superior frontal connections showed decreased connectivity in SWEDD compared to PD patients. These results are consistent with a recent MRI study [13, 26, 27]. The connections and related regions that were previously reported to have increased functional connectivity for PD patients are in the sensorimotor cortex, SMA, cingulate gyrus, primary motor cortex, and parietal cortex [13]. Similar connections and regions were identified using diffusion-weighted MRI in PD patients [27]. A previous study reported reduced fractional anisotropy (FA) values in the frontal lobe, premotor area, and cingulate in PD patients [26]. Among subcortical-cortical connections, seven showed significant group-wise differences. Pallidum-frontal pole, pallidum-postcentral gyrus, and putamen-precentral gyrus connections in the left hemisphere decreased in PD compared to SWEDD. Rostral middle frontal gyrus-putamen, rostral middle frontal gyrus-caudate, and rostral middle frontal gyrus-thalamus connections increased in PD compared to SWEDD. These results corroborate previous studies using fMRI and diffusion MRI in PD patients [8, 13]. A decrease in functional connectivity using fMRI was reported in the left putamen, SMA, and DLPFC in PD patients compared to NCs [13]. Other studies using diffusion MRI have shown that FA values in the putamen and caudate are lower in PD compared to NCs [8]. Within subcortical connections, five were significantly different between PD and SWEDD, in agreement with existing studies [8, 28–30]. Structural connectivity studies of PD patients have shown a reduction in connectivity in the nigrostriatal tract (connections among SN, STN, putamen, and pallidum) using DTI [28]. Others report decreased FA values in the substantia nigra, caudate, and putamen in PD patients [8]. One functional connectivity study showed a decreased connection in the pallidum-putamen using resting state functional MRI (rs-fMRI) [29]. PD patients also showed decreased fiber counts in SN-putamen and pallidum connections [30].

Existing connectivity studies have mainly explored differences between NCs and PD patients [8, 13, 26–31]. There is a lack of comparable studies comparing SWEDD and PD using structural connectivity. The previous discussion contrasting our findings with existing literature was largely derived from comparisons of PD patients and NCs, not necessarily PD and SWEDD. The existing literature describes various MRI techniques, including resting state fMRI, structural MRI, and diffusion MRI [8, 13, 26–31]. Comparisons between our findings and existing research should consider the imaging modalities used in those studies. Some researchers have adopted non-MRI and non-SPECT approaches to studying PD and SWEDD [32, 33]. One study successfully adopted electrophysiological tremor parameters to distinguish between tremor-dominant PD and SWEDD [32]. In addition, a sonography study attained 85% accuracy in distinguishing PD from SWEDD [33].

Our results showed significant connections between subcortical and cortical structures mostly in left intrahemisphere connections, as shown in Table 3. One possible explanation might be related to lateralized dopamine transporter uptake in PD. Previous studies using I^123^-IBZM SPECT revealed lateralized difference in the striatal uptake of I^123^-IBZM, which implies lateralized availability of dopamine-D2 receptors [34]. Another possible explanation might be the handedness of patients. Our study considered largely right-handed patients, which could be one factor leading to the lateralized connectivity patterns shown in this study. Some studies have found that right-handed PD patients have significantly lower dopamine transporter uptake in the left putamen compared to the right putamen [31]. Thus, lateralized connectivity differences in our study might be related to right-handed PD patients with reduced dopamine uptake in the left striatum. Side of disease onset in PD patients might differentially affect connectivity patterns [35, 36]. A previous study reported that an association between right-sided symptoms in PD and depression was related to the side of disease onset [37]. Unfortunately, the PPMI database lacked side of disease onset information; thus, we could not quantify the difference between sides of disease onset.

DaT imaging by definition can distinguish between PD and SWEDD [4, 16]. Our study showed that an MRI-based measure (i.e., DTI tractography) could also distinguish between PD and SWEDD. MRI has advantages over SPECT, as it is more readily available at clinical sites and does not expose patients to radiation. Our results using DTI showed better correlation with clinical variables such as MDS-UPDRS III. We reported a correlation value of 0.54 between DTI results and MDS-UPDRS, while correlation between DaT imaging measures (i.e., striatal binding ratio (SBR) of the caudate and putamen) and MDS-UPDRS from the same PPMI data showed correlation values ranging from −0.36 to −0.29, as shown in Table 6.

SWEDD patients are heterogeneous, with many different underlying conditions. Modern MRI techniques can quantify diffusion (as shown in DTI), perfusion, and other tissue properties [38–40]. Applying various MRI techniques to SWEDD patients could reveal differences that relate to various underlying conditions of SWEDD such as essential tremor and dystonic tremor. Unfortunately, the PPMI database simply did not have enough samples in the SWEDD group to subdivide them into different subgroups based on underlying condition. We leave this important topic for future research, which might be feasible as the PPMI collects more cases.

Our study has some limitations. First, DTI itself suffers from limitations including (1) inability to distinguish between efferent and afferent connections and (2) capacity to account for only major fiber tracts due to limited voxel resolution. Adopting an advanced form of DTI known as high-angular resolution diffusion imaging (HARDI) would allow for complex modeling within a voxel; however, HARDI requires longer scan times compared to DTI [41]. Second, multimodal imaging could be adopted to better explore the brain network. For example, we could have used additional fMRI to complement the DTI data [42]. Multimodal analysis of the brain network would allow incorporation of complementary information derived from different modalities in order to better quantify differences between PD and SWEDD. Third, our study only considered SWEDD and PD, leaving out normal controls. Future studies need to consider all three groups (i.e., PD, SWEDD, and NCs groups) so that the identity of SWEDD can be better established.

## 5. Conclusion

We identified four structural connections that distinguished between PD and SWEDD and also correlated well with MDS-UPDRS score. These findings confirm correlations between important connections and well-established clinical scores. Our study successfully distinguished between PD and SWEDD using these identified connections. Thus, our findings might serve as a first to step to investigate whether SWEDD patients have a unique connectivity profile.

## Figures and Tables

**Table 1:** Participant information: values are reported as mean ± standard deviation (SD).

	SWEDD	PD	*P* value
Number of subjects	*n* = 37	*n* = 40	—
Age (years)	61.3 ± 10.9	59.5 ± 11.0	0.403
Sex (male/female)	24/13	21/19	0.264
Disease duration (months)	6.4 ± 7.9	5.58 ± 4.7	0.587
Hoehn & Yahr score	1.6 ± 0.5	1.5 ± 0.5	0.921
MDS-UPDRS-III score	14.0 ± 9.1	18.6 ± 8.1	0.752
GDS score	3.3 ± 3.7	2.6 ± 3.1	0.392
MoCA score	27.4 ± 1.8	28.1 ± 2.1	0.103

**Table 2:** Motor-related regions that were explored for connectivity.

Cortical cluster	ROIs	Cortical cluster	ROIs
Motor, premotor cortex	Caudal middle frontal		Postcentral
	Precentral	Parietal lobe	Supramarginal
Dorsal lateral prefrontal cortex (DLPFC)	Frontal pole		Superior parietal
	Rostral middle frontal		Inferior parietal
	Pars triangularis		Thalamus proper
	Pars orbitalis		Caudate
Cingulate motor area (CMA)	Rostral anterior cingulate	Subcortical	Putamen
	Posterior cingulate		Pallidum
Supplementary motor area (SMA)	Paracentral		Accumbens area
	Superior frontal	Brainstem	Brainstem

**Table 3:** Identified connections with significant group-wise differences. Significantly different connections are reported in terms of connectivity values and corrected *P* values. Connectivity values are reported as mean ± standard deviation (SD). Bold text shows identified connections with significant differences (corrected *P* < 0.001) between PD and SWEDD.

	Group-wise differences			
	Connection	Number of fibers (mean ± SD)		Corrected *P* value
		PD	SWEDD	
(a) Cortico-cortical connection	L_precentral-L_posteriorcingulate	4.6 ± 8.23	1.56 ± 2.89	0.028
	L_paracentral-L_superiorfrontal	1.18 ± 1.67	3.21 ± 6.29	0.039
	**L_paracentral-L_posteriorcingulate**	**8.72 ± 8.32**	**3.48 ± 4.35**	**<0.001**
	L_posteriorcingulate-L_superiorfrontal	11.7 ± 8.96	6.27 ± 6.23	0.003
	L_parsorbitalis-R_superiorfrontal	0.65 ± 3.33	2.18 ± 4.72	0.046
	R_paracentral-L_paracentral	0.65 ± 2.02	2.59 ± 4.5	0.014
	**R_paracentral-R_posteriorcingulate**	**37.7 ± 23.47**	**17.8 ± 23.3**	**<0.001**
	R_precentral-R_posteriorcingulate	13.1 ± 16.75	5.54 ± 14.3	0.034
	**R_posteriorcingulate-R_superiorfrontal**	**43.67 ± 27.54**	**19.94 ± 21.7**	**<0.001**
	R_posteriorcingulate-R_superiorparietal	10.45 ± 17.27	3.00 ±6.78	0.005
	R_rostralmiddlefrontal-R_inferiorparietal	1.42 ± 3.20	0.29 ± 1.17	0.045

(b) Cortico-subcortical connection	L_pallidum-L_frontalpole	0.77 ± 1.51	2.83 ± 6.40	0.045
	L_pallidum-L_postcentral	5.52 ± 9.17	16.08 ± 26.3	0.012
	L_putamen-L_precentral	14.5 ± 8.95	24.86 ± 24.48	0.013
	L_putamen-L_rostralmiddlefrontal	34.87 ± 30.86	21.83 ± 21.34	0.035
	L_caudate-L_rostralmiddlefrontal	37.5 ± 41.4	15.59 ± 24.66	0.027
	L_thalamusproper-L_rostralmiddlefrontal	7.27 ± 9.75	3.27 ± 5.02	0.007
	R_pallidum-R_superiorfrontal	1.50 ± 2.76	4.57 ± 7.97	0.022

(c) Subcortical connection	L_caudate-L_putamen	2.28 ± 3.34	5.59 ± 7.58	0.009
	R_putamen-R_pallidum	2.28 ± 2.33	4.51 ± 3.98	0.002
	Brainstem-L_putamen	3.80 ± 5.71	7.78 ± 10.96	0.046
	Brainstem-L_caudate	2.00 ± 4.29	4.51 ± 6.46	0.048
	Brainstem-R_thalamusproper	31.48 ± 18.72	42.08 ± 23.13	0.029

**Table 4:** Correlation with clinical scores was investigated. Structural connectivity values based on the number of fibers connecting two regions are reported within the identified connections. Correlations between structural connectivity and MDS-UPDSR score are reported as Spearman correlation coefficients, with corrected *P* values in the rightmost column. Bold text shows the connections that significantly correlated with MDS-UPDRS-III (corrected *P* < 0.001).

	Correlation with MDS-UPDRS-III score				
	Connection	Number of fibers (mean ± SD)			Corr coef. (corrected *P* value)
		PD	SWEDD	Total	
(a) Cortico-cortical connection	L_precentral-L_posteriorcingulate	4.6 ± 8.23	1.56 ± 2.89	3.14 ± 6.39	0.16 (1)
	**L_paracentral-L_superiorfrontal**	**1.18 ± 1.67**	**3.21 ± 6.29**	**2.15 ± 4.61**	**−0.32 (<0.001)**
	L_paracentral-L_posteriorcingulate	8.72 ± 8.32	3.48 ± 4.35	6.21 ± 7.17	0.15 (1)
	L_posteriorcingulate-L_superiorfrontal	11.7 ± 8.96	6.27 ± 6.23	9.09 ± 8.20	0.04 (1)
	L_parsorbitalis-R_superiorfrontal	0.65 ± 3.33	2.18 ± 4.72	1.39 ± 4.10	0.01 (1)
	R_paracentral-L_paracentral	0.65 ± 2.02	2.59 ± 4.5	1.58 ± 5.56	0.03 (1)
	**R_paracentral-R_posteriorcingulate**	**37.7 ± 23.47**	**17.8 ± 23.3**	**28.19 ± 25.33**	**0.56 (<0.001)**
	R_precentral-R_posteriorcingulate	13.1 ± 16.75	5.54 ± 14.3	9.47 ± 15.98	0.17 (0.992)
	**R_posteriorcingulate-R_superiorfrontal**	**43.67 ± 27.54**	**19.94 ± 21.7**	**32.27 ± 27.48**	**0.54 (<0.001)**
	R_posteriorcingulate-R_superiorparietal	10.45 ± 17.27	3.00 ± 6.78	6.87 ± 13.74	0.27 (0.057)
	R_rostralmiddlefrontal-R_inferiorparietal	1.42 ± 3.20	0.29 ± 1.17	0.88 ± 2.50	0.03 (1)

(b) Cortico-subcortical connection	L_pallidum-L_frontalpole	0.77 ± 1.51	2.83 ± 6.40	1.76 ± 4.65	−0.04 (1)
	L_pallidum-L_postcentral	5.52 ± 9.17	16.08 ± 26.3	10.60 ± 19.98	0.18 (0.85)
	**L_putamen-L_precentral**	**14.5 ± 8.95**	**24.86 ± 24.48**	**19.48 ± 18.77**	**0.54 (<0.001)**
	L_putamen-L_rostralmiddlefrontal	34.87 ± 30.86	21.83 ± 21.34	28.61 ± 27.34	0.09 (1)
	L_caudate-L_rostralmiddlefrontal	37.5 ± 41.4	15.59 ± 24.66	26.97 ± 35.90	−0.08 (1)
	L_thalamusproper-L_rostralmiddlefrontal	7.27 ± 9.75	3.27 ± 5.02	5.35 ± 8.05	0.02 (1)
	R_pallidum-R_superiorfrontal	1.50 ± 2.76	4.57 ± 7.97	2.97 ± 6.03	0.01 (1)

(c) Subcortical connection	L_caudate-L_putamen	2.28 ± 3.34	5.59 ± 7.58	3.7 ± 5.98	0.06 (1)
	R_putamen-R_pallidum	2.28 ± 2.33	4.51 ± 3.98	3.35 ± 3.40	−0.14 (1)
	Brainstem-L_putamen	3.80 ± 5.71	7.78 ± 10.96	5.71 ± 8.81	0.15 (1)
	Brainstem-L_caudate	2.00 ± 4.29	4.51 ± 6.46	3.21 ± 5.55	0.01 (1)
	Brainstem-R_thalamusproper	31.48 ± 18.72	42.08 ± 23.13	36.57 ± 21.49	0.19 (0.648)

**Table 5:** Classifier performance to distinguish between PD and SWEDD.

Group	Sensitivity (%)	Specificity (%)	Accuracy (%)
SWEDD versus PD	78.38	77.50	77.92

**Table 6:** Correlations between DaT imaging measures and MDS-UPDRS score.

	Left caudate SBR	Left putamen SBR	Right caudate SBR	Right putamen SBR
*R*-value	−0.33	−0.33	−0.29	−0.36
*P* value	0.003	0.003	0.011	0.001
